# Environmental perceptions and objective walking trail audits inform a community-based participatory research walking intervention

**DOI:** 10.1186/1479-5868-9-6

**Published:** 2012-01-30

**Authors:** Jamie Zoellner, Jennie L Hill, Karen Zynda, Alicia D Sample, Kathleen Yadrick

**Affiliations:** 1 Department of Human Nutrition, Foods and Exercise, Virginia Tech, 1981 Kraft Drive (0913), Blacksburg, VA 24061, USA; 2 Department of Nutrition and Food Systems, The University of Southern Mississippi, 118 College Drive Box #5172, Hattiesburg, MS 39406-0001, USA

## Abstract

**Background:**

Given the documented physical activity disparities that exist among low-income minority communities and the increased focused on socio-ecological approaches to address physical inactivity, efforts aimed at understanding the built environment to support physical activity are needed. This community-based participatory research (CBPR) project investigates walking trails perceptions in a high minority southern community and objectively examines walking trails. The primary aim is to explore if perceived and objective audit variables predict meeting recommendations for walking and physical activity, MET/minutes/week of physical activity, and frequency of trail use.

**Methods:**

A proportional sampling plan was used to survey community residents in this cross-sectional study. Previously validated instruments were pilot tested and appropriately adapted and included the short version of the validated International Physical Activity Questionnaire, trail use, and perceptions of walking trails. Walking trails were assessed using the valid and reliable Path Environmental Audit Tool which assesses four content areas including: design features, amenities, maintenance, and pedestrian safety from traffic. Analyses included Chi-square, one-way ANOVA's, multiple linear regression, and multiple logistic models.

**Results:**

Numerous (n = 21) high quality walking trails were available. Across trails, there were very few indicators of incivilities and safety features rated relatively high. Among the 372 respondents, trail use significantly predicted meeting recommendations for walking and physical activity, and MET/minutes/week. While controlling for other variables, significant predictors of trail use included proximity to trails, as well as perceptions of walking trail safety, trail amenities, and neighborhood pedestrian safety. Furthermore, while controlling for education, gender, and income; for every one time per week increase in using walking trails, the odds for meeting walking recommendations increased 1.27 times, and the odds for meeting PA recommendation increased 3.54 times. Perceived and objective audit variables did not predict meeting physical activity recommendations.

**Conclusions:**

To improve physical activity levels, intervention efforts are needed to maximize the use of existing trails, as well as improve residents' perceptions related to incivilities, safety, conditions of trail, and amenities of the walking trails. This study provides important insights for informing development of the CBPR walking intervention and informing local recreational and environmental policies in this southern community.

## Background

Major risk factors for cardiovascular disease (CVD) include hypertension (HTN), lack of physical activity, and overweight/obesity. Unfortunately, racial/ethnic disparities exist for all of these risk factors. Specific to hypertension, national data indicates that the age-adjusted prevalence of hypertension has not changed significantly in the past 10 years, and that substantial (> 10%) racial/ethnic disparities persist [1,2]. In fact, estimated at 42.0%, non-Hispanic blacks persistently have the highest age-adjusted prevalence of hypertension as compared to 28.8% among non-Hispanic whites and 25.5% among Mexican Americans [1,2]. In addition to racial/ethnic health disparities, regional and state health disparities also continue. When compared to national averages, epidemiological data consistently indicate that adults in Mississippi are more likely to have hypertension (i.e. ranks 49^th^), high cholesterol (i.e. ranks 48^th^), obesity (i.e. ranks 50^th^), and among the least likely to meet physical activity recommendations (i.e. ranks 49^th^)[3]. Based on these discouraging prevalence data, it is no surprise that Mississippi's CVD mortality is the highest in the nation [3], and CVD is the leading cause of death in Mississippi [4]. While a variety of individual, social, environmental, and policy factors likely contribute to these rankings [5-7], the research reported here focuses on the physical activity patterns and the availability of walking trails supportive of physical activity promotion.

Despite the well-established protective effects of physical activity on all cause mortality and CVD risk factors, most in the U.S. are physically inactive [8]. Although racial/ethnicity disparities for recreational physical activity persist, some evidence suggests that walking is the preferred method of exercise among low-income African-Americans and that they are more likely to use outdoor activity facilities (e.g. walking trails) [9]. Given the documented physical activity disparities that exist among low-income minority communities and the increased focused on socio-ecological approaches to address physical inactivity, examination of how the built environment supports physical activity has received heightened attention in recent years [10-17].

Numerous factors should be taken into consideration when investigating environmental correlates with physical activity levels. Two reviews have found a positive relationship among proximity to walking trails, tracks, and other recreational facilities with physical activity levels [18,19]. While several studies have examined physical activity resources, and access inequities associated with racial diversity and low socioeconomic status have emerged, the results have been inconsistent [10,20-24]. Even if infrastructure does exist, perceptions of or actual crime, litter, graffiti, and other deterrents have been shown to decrease accessibility and use of public recreational environments [25,26]. Collectively, the burgeoning body of scientific literature highlights the importance of understanding proximity to physical activity outlets and both objective and perceived measures of the built environment to support physical activity [18,25,27]. This information is especially useful as it relates to the development of culturally appropriate interventions and informing local recreational and environmental policies for health disparate communities [13].

The current study is part of a community-based participatory research (CBPR) walking intervention, *Healthy U Begins with (H.U.B.) City Steps*, designed to promote physical activity and build community capacity to achieve a sustained reduction in blood pressure among African American adults in Hattiesburg, Mississippi [28,29]. The CBPR research approach is designed to ensure community participation in all aspects of the research process [30,31]. Promoting co-learning and capacity-building among partners is a fundamental principle of CBPR, as are shared decision-making power and mutual ownership of the research process and products [32,33]. Importantly, applying CBPR principles to development and execution of environmental physical activity studies has been a recent key recommendation [13]. In the context of the larger CBPR walking intervention, this study was conducted to inform development of the *H.U.B. City Steps *intervention, to provide baseline data for assessing changes in social and environmental phenomena related to physical activity, to engage and train local community members in the survey research process, and to develop a community resource guide intended to highlight and promote walking trails for intervention participants and local stakeholders.

This paper explores physical activity and trail use patterns and investigates individual perceptions of walking trail and neighborhood characteristics among adults in Hattiesburg, Mississippi, and examines objective characteristics of walking trails. The primary aim is to explore if perceived and objective audit variables predict meeting recommendations for walking and physical activity, MET/minutes/week of physical activity, and frequency of trail use.

## Methods

### Community-based participatory research approach

This research was approved by the Institutional Review Board at the University of Southern Mississippi. The *H.U.B. City Steps *Community Advisory Board (CAB) and local community members participated in the development, implementation and evaluation of this research project. The CAB provided feedback on the cultural sensitivity of the perceptions survey instrument and nominated community members to serve as data collectors. Fourteen lay community members were hired and trained to interview-administer the perceptions survey. These community members were compensated for their participation in the research project. Two community research staff members were also trained to participate in the path audits.

### Targeted population

The target population included adults residing in Hattiesburg, Mississippi. Hattiesburg is located in southeast Mississippi, and has a population of approximately 45,000 residents. The median household income of Hattiesburg is $24,409, which is lower than state and national averages at $31,330 and $41,994, respectively [34]. The city is approximately 47% African-American and 49% White. Twenty-four percent of deaths among non-whites in 2007 in Hattiesburg were attributable to heart disease, cerebrovascular disease, and stroke, but no incidence or prevalence data are available for these or other conditions at the city or county level[35]. For non-whites in the nine-county southeast Mississippi public health district in which Hattiesburg is located, the prevalence rate for hypertension in 2007 was 43% [36].

### Participant recruitment

Eligibility criteria included 18 years of age or older and residing within the city of Hattiesburg. A proportional sampling plan was developed to match the targeted enrollment matrix for the larger CBPR walking intervention. Specifically, the goal was to sample 75-80% African Americans and 20-25% whites; and approximately 50% men and 50% women. The 14 community interviewers attended a one-day group training workshop, and passed a certification exercise at an individual follow-up session. The community data collectors recruited participants according to the race and gender sampling matrix. The primary recruitment method was word of mouth through the data collectors' established neighborhood and social networks. The interview-administered surveys were completed between July-September 2009, and on average took 25 minutes to complete. Respondents received a $10 gift card.

### Instrument development and pilot testing

Previously validated instruments were utilized and/or adapted to meet the needs of this CBPR project [21,37]. Content validity of the survey instrument was established by an expert panel review (n = 6). One of the most notable changes was reformatting numerous single questions allowing for multiple responses into statements on a Likert scale to improve the richness of responses. In addition to the expert panel, members of the CBPR intervention CAB also provided valuable feedback to improve the cultural sensitivity of the survey instrument. Prior to use in this study, the survey instrument was pilot tested using a self-administered method within a sample of 92 women, primarily African American (85.9%). While some minor content and formatting changes resulted from this pilot test, the most notable modification was changing the methods from a self-administered format to an interview-administered format due to the large amount of missing data and skipped survey questions in the pilot study.

### Physical activity measures

The survey included the short version of the validated International Physical Activity Questionnaire (IPAQ) to assess participants' activity levels [37]. Participants reported the number of days per week and amount of time spent per day during the past seven days in three types of activity: vigorous activity, moderate activity, and walking. Using standardized scoring procedures, this information was used to create three outcome variables for respondents. First, to classify walking behavior in a manner similar to the Centers for Disease Control and Prevention, we computed a dichotomous variable (yes/no) for 'meeting recommendations for physical activity by walking,' defined as walking 5 or more days per week for at least 30 minutes each day [38,39]. 'Meeting recommendations for physical activity' is a dichotomous variable (yes/no) scored according to published IPAQ procedures and includes walking and moderate to vigorous physical activity done five or more days per week for at least 30 minutes [37]. Lastly, we computed a continuous score for total physical activity metabolic equivalent (MET)/minutes/week (sum of walking + moderate + vigorous MET/minutes/week scores) [37]. The IPAQ is one of the most commonly used self-reported physical activity instruments for population surveillance among adults. The IPAQ is reliable (α = 0.8) [40], has shown modest validity when compared to accelerometers (r = 0.3) [40], and has been used in low socioeconomic and multiethnic populations [41,42].

### Trail use

Participants reported the name of and the distance it took to travel to the trail they used most often. The frequency (never, 1-3 times per month, 1-2 times per week, 3-4 times per week, 5-6 times per week, or 1 time per day) at which they used walking trails during the winter, spring, summer and fall seasons was reported. From individual season estimates, a weekly frequency for trail use was created.

### Perceptions of neighborhood and walking trails

Perceptions of walking trails and the neighborhood environment, and barriers and enablers for physical activity use, were adapted from a telephone-administered survey from Brownson and colleagues [21]. Participants responded to 15 statements regarding the walking trail they used most often and 13 statements regarding the walking environment within their neighborhood (1 = strongly disagree, 4 = strongly agree). For example, walking trail statements related to lighting, safety and crime, litter, stray animals, parking, restrooms, beauty, crowds, availability of benches, and overall satisfaction with the trail. Neighborhood statements related to the availability and maintenance of sidewalks, hills, beauty, traffic and exhaust fumes, lighting, loose dogs, and safety and crime during the day and night.

Using methods based on published protocols for similar instruments [43], four summary scores for perception of safety in neighborhoods and on trails were computed from the individual perceptions data. Items were grouped based on scoring for similar measures. Further, exploratory factor analyses (EFA) were conducted to ensure that items were placed in a single index score. Reliability statistics are provided for each computed scale. Perceptions of pedestrian safety included six items, presence and maintenance of sidewalks, lighting, presence of dogs, speed of traffic and safety from traffic (Cronbach Alpha = 0.66). Safety from crime in the neighborhood included two items about safety from crime during the day and in the evening (Cronbach alpha = 0.82). Perception of the safety of the trail included five items such as feeling safe on the trail, crime on the trail, presence of lighting, condition of trail surface, and presence of animals (Cronbach alpha = 0.68). Finally, trail amenities included seven items such as aesthetics of trail, fitness or exercise equipment, restrooms, and rest stops (e.g. benches, gazebos and picnic benches) (Cronbach Alpha = 0.83).

### Demographics

Demographic variables included gender, race and ethnicity, age, and self-reported height and weight. Income was reported across 12 categories of $5,000 increments and collapsed to three categories for analyses. Similarly education level was reported across nine categories and collapsed to three categories for analyses.

### Path Environmental Audit Tool (PEAT)

Within four city wards targeted by the CBPR intervention, a total of 21 walking trails were identified via a local running/walk club website, a brochure of walking trails created by the city department of parks and recreation, and through discussions with community members. Walking trails were assessed using the valid and reliable Path Environmental Audit Tool (PEAT) [44,45]. The PEAT assesses four content areas including: design features, amenities, maintenance, and pedestrian safety from traffic (e.g. roads that intersect trail, pedestrian crossings marked). Since gazebos are recognized as an important feature of walking trails and parks in the South, the only adaptation made to the PEAT instrument was adding gazebos as an amenity along with wheelchair accessibility to the gazebo.

Three research staff members and two community project staff members were trained using the PEAT manual [45]. Training involved didactic review and discussion of the manual, followed by team visits to two trails where coders independently assessed the trail and then met to discuss and resolve uncertainty. Trail assessments took place between February-July 2009 and each trail was assessed by at least two trained auditors. Photographs were also taken for development of the community resource guide and to document the various design, amenities, and maintenance features of the trail.

We assessed quality of audits by examining consistency of auditors across trails and inter-rater reliability for each trail. Auditors conducted trail assessments in pairs or in threes. One-way ANOVAs or t-tests indicated there were no consistent differences between possible combinations of auditors. The audit tool had 98 items total. Inter-rater reliability of the instrument was assessed by computing a kappa coefficient for a subset of items (n = 16) that included items from each of the four primary content areas measured by the PEAT instrument [46]. Using previously established criteria in which a kappa value of > 0.60 = 'good to excellent', 0.41-0.60 = 'moderate' and < 0.40 = 'poor'; good to excellent inter-rater reliability for the audit tool was confirmed. Computed kappa values ranged from 0.35-1.0 across the trails with a mean of 0.77.

### Data Analysis

SPSS version 18 was used for data analysis. Descriptive statistics including frequencies, percents, means, and standard deviations were used to summarize all data. Internal reliability statistics were conducted on all scales and Cronbach alphas are provided. Chi-square and one-way ANOVA's were used to explore associations among demographic variables and meeting recommendations for walking and physical activity, MET/minutes/week of physical activity, and frequency of trail use.

Given the high inter-rater reliability for the PEAT data, a random number table was generated to reduce the dataset to a single audit for analytic purposes. For analytic purposes, and modeled after scoring procedures for the Physical Activity Resource Assessment (PARA) instrument [47], standardized means were computed to represent scales that captured overall features of the trails including incivilities (e.g. graffiti, vandalism, litter, trash, odors, noise), amenities (e.g. restrooms, water fountains, benches, gazebos, places to purchase food, etc), safety (e.g. safety of intersections with traffic, buffer from roadway, call box, site distance) and condition of the trail (e.g. surface, slope and grade, points of interest, etc.). A standardized mean score close to zero indicates low encounters (few of the item existed), whereas a score closer to 1.0 indicates more or frequent encounters.

Multiple linear regression and multiple logistic models were used to test the following hypotheses: 1) increased trail use would positively predict meeting recommendations for walking and physical activity, as well at total MET/minutes/week of physical activity; 2) proximity to the trail would positively predict meeting recommendations for walking and physical activity, MET/minutes/week of physical activity, and frequency of trail use; 3) positive perceptions of the neighborhood and trails would positively predict meeting walking and physical activity recommendations, MET/minutes/week of physical activity, and frequency of trail use; and 4) objective audits of walking trails would positively predict meeting recommendations for walking and physical activity, MET/minutes/week of physical activity, and frequency of trail use. When exploring the ability of objective audit variables assessed with the PEAT tool to predict physical activity behaviors, individuals were matched with audited trail data from the trail they indicated using most often. Purposeful selection methods were used to enter and test potential covariates into the model. The final set of significant covariates, including education, gender, and income, are used in all models for all outcomes for consistency. Standardized coefficients are reported, as well as p-values at the < 0.05 and < 0.01 levels.

## Results

### Demographic characteristics, physical activity patterns, and trail use pattern

The demographics and activity level of participants are illustrated in Table 1. The goal to sample 75-80% African Americans and 20-25% whites, and approximately 50% men and 50% women was sufficiently achieved. In the overall sample, 35% of respondents met walking recommendations, 57% met PA recommendations, and the average MET/minutes/week was 4141 (SD = 3887). As compared to women, men were significantly more likely to meet walking recommendations, PA recommendation, and achieve more MET/minutes/week. Respondents with a college education reported significantly lower MET/minutes/week as compared with the other education categories, and respondents earning < $19,000 reported significantly more MET/minutes when compared with the two higher income categories. When asked to select their top two choices for physical activity, 61% indicated walking, followed by housework (38%), weight lifting (17%), and gardening or yard work (13%). When averaged across the seasons, mean trail use was 1.5 (SD = 1.6) times per week, which included approximately 18%, 33%, 28%, 17% and 4% of respondents who used the trails never, 1-3 times per month, 1-2 times per week, 3-4 times per week, and 5 or more times per week. As further indicated in Table 1, trail use did not vary significantly by any demographic characteristics.

**Table 1:** Characteristics of respondents in relation to meeting physical activity recommendations by walking, meeting physical activity recommendations, average MET/min, and trail use (n = 372)

Demographic category	N (%)	**N (%) within category that meets PA recommendation by walking**^a, b^	**N (%) within category that meets recommendation for PA**^a, c^	**Mean (SD) MET/min**^a, d^	Mean (SD) days of weekly trail use
**Gender**					
Female	202 (54%)	63 (31%)*	102 (50%)**	3443 (3402) **	1.4 (1.5)
Male	170 (46%)	66 (39%)*	111 (65%)**	4992 (4264) **	1.5 (1.7)

**Race**					
African American	293 (79%)	104 (35%)	171(58%)	4099 (3844)	1.4 (1.5)
Caucasian	61(16%)	22 (36%)	35 (57%)	4405 (4192)	1.6 (1.8)
Other	9 (2%)	2 (22%)	7 (78%)	4068 (3530)	1.5 (1.5)

**Age**					
18-30 years	144 (39%)	46 (32%)	82 (57%)	3883 (3697)	1.5 (1.6)
31-40 years	105 (28%)	40 (38%)	68 (65%)	4551 (4375)	1.1 (1.2)
41-50 years	65 (17%)	21 (32%)	38 (58%)	4035 (3811)	1.6 (1.7)
51-60 years	33 (9%)	15 (45%)	17 (51%)	3998 (3249)	1.6 (2.1)
60 years or older	17 (5%)	7 (41%)	5 (29%)	3851 (3800)	1.3 (1.9)

**Highest Level of School**					
6^th ^-12^th ^grade or HS diploma	101 (27%)	37 (37%)	57 (56%)	4851 (4666)*	1.4 (1.7)
Trade school or some college	131 (35%)	44 (34%)	74 (56%)	4327 (3751)*	1.3 (1.6)
College degree	139 (37%)	47 (34%)	82 (59%)	3467 (3284)*	1.6 (1.5)

**Income level**					
≤ $19,999	109 (29%)	34 (31%)	71 (65%)	5034 (4593)*	1.5 (1.7)
$20,000-39,999	116 (31%)	37 (32%)	62 (53%)	3759 (3694)*	1.5 (1.5)
≥ $40,000	92 (25%)	39 (42%)	53 (58%)	3919 (3128)*	1.2 (1.6)

**Body Mass Index**					
Underweight	3 (1%)	1 (33%)	1 (33%)	5004 (8667)	0.01 (0.02)
Healthy Weight	96 (26%)	32 (33%)	51 (53%)	4029 (3960)	1.4 (1.7)
Overweight	131 (35%)	40 (31%)	80 (61%)	4075 (3821)	1.5 (1.6)
Obese	109 (29%)	43 (39%)	66 (61%)	4471 (3739)	1.3 (1.5)

*p-value < 0.05 within demographic category; ** p < 0.01 within demographic category

^a ^Assessed using the short version of International Physical Activity Questionnaire (IPAQ)

^b ^Dichotomized variable (yes/no) for meeting walking recommendations of 5 or more days of walking lasting at least 30 minutes

^c ^Dichotomized variable (yes/no) for meeting physical activity recommendations of 5 or more days of moderate physical activity lasting at least 30 minutes

^d^Continuous variable for total physical activity MET-minutes/week (sum of walking + moderate + vigorous MET- minutes/week scores)

### Results of the Path Environmental Audit Tool (PEAT)

Eight of the 21 tracks were either owned by the county, university, or a healthcare institution and the remaining 13 were owned and maintained by the city of Hattiesburg. Of the 21 trails, 19 were circular, 1 was semi-circular, and 1 was a Rails to Trails Conservancy initiative. As illustrated in Table 2, and as indicated by the low standardized mean of 0.11 (SD = 0.12), there were very few indicators of incivilities (e.g. graffiti, litter, etc) across all trails. In general, safety features rated relatively high across the trails with a mean of 0.64 (SD = 0.15). The trails were primarily pedestrian areas with only one trail intersecting with a roadway. The overall standardized mean of 0.17 (SD = 0.09) for maintenance and aesthetics of the trail was low. Despite the overall low standardized mean, the standardized mean of the single item for condition of path was 0.76 and 52% of trails had good or excellent surface conditions. The overall low standardized mean is attributed to the finding that the trails generally lacked points of visual interest or aesthetic appeal and 84% of the trails were predominantly flat or gently sloping. As indicated by the standardized mean of 0.42 (SD = 0.14), amenities varied across the trails. Benches and gazebos were common amenities, and 92% had some level of signage along the trail and adequate parking for cars at the trail entrance or nearby. However, only about 50% had restrooms, and very few trails offered services such as food or access to other community venues, civic institutions (e.g. schools, museums, and churches) or commercial venues.

**Table 2:** Summary data for Path Environmental Audit Tool (PEAT) audit tool (n = 21 trails)

Audit Summary Scales	**Mean (SD)**^a^	Example of items included from PEAT	Present/Rating
Incivilities	0.11 (0.12)	• Graffiti, vandalism • Litter, Trash	20% some or a lot 16% some or a lot

Safety	0.64 (0.15)	• Buffer from roadway • Wide buffer	88% w/buffer 88% > 3 foot buffer

Condition of Trail (includes maintenance & aesthetics)	0.17 (0.09)	• Overall condition of trail/path • Slope and grade of trail	52% good- excellent 84% flat or gentle slope

Amenities	0.42 (0.14)	• Restrooms • Benches • Gazebos	52% present (65% good-excellent condition) 40% present (65% good-excellent condition) 56% present (71% good-excellent condition)

^a^scores are standardized, scores close to zero would indicate a low amount or that very few of the item existed whereas closer to 1.0 indicates a lot or frequent encounters

### Trail use to predict walking, physical activity, and MET/minutes/week of physical activity

As illustrated in Table 3, trail use significantly predicted meeting recommendations for walking, for physical activity, and MET/minutes/week of physical activity. For example, while controlling for education, gender, and income; for every one time per week increase in using walking trails, the odds for meeting walking recommendations increased 1.27 times, and the odds for meeting PA recommendation increased 3.54 times. Further, the number of MET/minutes/week of physical activity increased 0.23 standard deviations for every one time per week of using walking trails, while accounting for other demographic variables.

**Table 3:** Weekly trail use predicts meeting physical activity recommendations by walking, meeting physical activity recommendations, and MET/MINs of physical activity (n = 372)

	Meeting PA recommendations **by walking**^a, b, e^		Meeting PA **recommendations**^a, c, e^		MET/MINs **of PA **^a, d, e^
	**β (SE)**	**OR (95% CI)**	**β (SE)**	**OR (95% CI)**	**β (SE)**

**Weekly trail use**	.242 (.28)**	1.27 (1.09-1.49)	1.26 (.10)**	3.54 (1.37-2.02)	.225 (143.77) **

*p-value < 0.05; ** p < 0.01

^a^Assessed using the short version of International Physical Activity Questionnaire (IPAQ)

^b^Dichotomized variable (yes/no) for meeting walking recommendations of 5 or more days of walking lasting at least 30 minutes

^c^Dichotomized variable (yes/no) for meeting physical activity recommendations of 5 or more days of moderate physical activity lasting at least 30 minutes

^d^Continuous variable for total physical activity MET-minutes/week (sum of walking + moderate + vigorous MET- minutes/week scores)

^e^Controls for education, gender, and income

### Proximity to the trails to predict walking, physical activity, MET/MINs of physical activity, and weekly trail use

Twenty-eight percent of the sample lived < 1 mile from a trail, 20% lived 1-4 miles away from a trail, and 52% lived ≥ 5 miles away from a trail. Proximity to trails did not predict meeting recommendations for walking or physical activity (Table 4), or MET/minutes/week of physical activity (MET/min data not shown). Conversely, while controlling for demographic variables, respondents who resided ≥ 5 miles to a walking trail were significantly less likely to use the trail.

**Table 4:** Logistic regression and linear models for predicting meeting physical activity recommendations by walking, meeting physical activity recommendations, and trail use (n = 372)

	Meeting PA recommendations **by walking**^a, b, e^		**Meeting PA recommendations**^a, c, e^		**Weekly Trail Use**^d, e^
	**β (SE)**	**OR (95% CI)**	**β (SE)**	**OR (95% CI)**	**β (SE)**

**Distance**					
Distance to Trail (ref = < 1 mile)					
2-4 miles	-0.09 (0.29)	0.92 (.52-1.62)	0.21 (0.30)	1.24 (.69-2.24)	-.09 (.22)
≥ 5 miles	-0.19 (0.44)	0.83 (.35-1.96)	0.53 (0.46)	1.70 (.69-4.15)	-.16 (.22)*

**Perceived Variables**	**β (SE)**	**OR (95% CI)**	**β (SE)**	**OR (95% CI)**	**β (SE)**
Pedestrian safety	-0.23 (0.18)	0.79 (.55-1.13)	-0.09 (0.18)	0.91 (.64-1.28)	.18 (.13)**
Safety from crime	-0.53 (0.14)	0.95 (.72-1.25)	0.20 (0.14)	1.22 (.92-1.62)	.05 (.11)
Trail safety	-0.17 (0.19)	0.84 (.58-1.22)	-0.22 (0.19)	0.98 (.67-1.43)	.16 (.14)**
Trail amenities	-0.01 (0.20)	0.99 (.68-1.45)	0.29 (0.20)	1.33 (.90-1.97)	.13 (.14)*

**PEAT Variables**	**β (SE)**	**OR (95% CI)**	**β (SE)**	**OR (95% CI)**	**β (SE)**
Incivilities	1.67 (2.22)	5.31(.67-409.9)	0.02 (2.27)	1.02 (.12-8.2)	-.11 (1.51)
Safety	-1.39 (1.31)	0.25 (.19-3.26)	-2.31 (1.55)	0.1 (.05-2.0)	-.01 (0.90)
Condition of trail	0.46 (2.34)	1.59 (.16-156.3)	0.58 (2.42)	1.79 (.16-204.9)	.13 (1.96)
Amenities	0.77 (2.03)	2.15 (.40-115.5)	3.16 (2.03)	23.58 (.44-263.3)	.10 (1.55)

*p-value < 0.05; ** p < 0.01

^a^Assessed using the short version of International Physical Activity Questionnaire (IPAQ)

^b^Dichotomized variable (yes/no) for meeting walking recommendations of 5 or more days of walking lasting at least 30 minutes

^c^Dichotomized variable (yes/no) for meeting physical activity recommendations of 5 or more days of moderate physical activity lasting at least 30 minutes

^d^Weekly frequency for trail use, averaged across four seasons

^e^Controls for education, gender, and income

### Perceptions of the environment to predict walking, physical activity, MET/minutes/week of physical activity, and weekly trail use

On a 4-point Likert scale (1 = strongly disagree, 4 = strongly agree), perceptions of pedestrian safety was 2.4 (SD = 0.7), safety from crime was 3.2 (SD = 0.9), overall perceptions of the walking trail safety averaged 3.0 (SD = 0.7), and trail amenities was 2.7 (SD = 0.7). None of these environmental perception variables predicted meeting recommendations for walking or physical activity (Table 4) or MET/minutes/week of physical activity (MET/min data not shown). However, the frequency of trail use increased with higher perceptions of pedestrian safety, trail safety, and trail amenities.

### Objective audit indicators to predict walking, physical activity, MET/minutes/week of physical activity, and weekly trail use

For testing objective audit indicators gathered with the PEAT tool, individuals were matched with audited trail data from the trail they indicated using most often. None of the objective indicators including incivilities, safety, condition of trails, or amenities were predictive of any of the hypothesized dependent indicators of physical activity and trail use (Table 4). Due to limitations with the standardized mean score for conditions of trail (maintenance and aesthetics of the trails), the regression models were run using the single item indicator for path condition. However, this did not produce any meaningful or significant changes in the objective audit regression models.

## Discussion

Given the emphasis and emerging importance of the built environment on influencing physical activity [10-17], this study provides important insights for informing development of the CBPR walking intervention and informing local recreational and environmental policies in the Hattiesburg region. In this study, we found that proximity to trails as well as higher perceptions of pedestrian safety, trail safety, and trail amenities predicted frequency of trail use. Further, we found that weekly trail use positively predicted the odds of meeting walking recommendations, physical activity recommendation, and total MET/minutes per week. Our findings support previous findings that indicate a relationship between trail proximity, frequency of trail use, and physical activity [48-50].

We did not find a relationship between objective audits and meeting walking or PA recommendations, nor did we find that perceptions of the environment predicted meeting walking or PA recommendations. While previous studies have found that perceived and objective environmental measures relate to physical activity differently [51-53], our study provides no evidence that either was a good predictor of physical activity. Despite the increased focus on the physical environment in recent years, relatively few studies have used objective, in-person audits to understand the environmental influence on meeting physical activity recommendations [27,54,55]. For examples, Zenk and colleagues found no association between objectively measured environmental variables and adherence to a 12-month walking intervention [55]. However, Lee and colleagues found that lower speed limits were most commonly associated with increased physical activity among both women and men [27]. Yet, contrary to hypotheses, greater segment connectivity was not associated with more physical activity [27]. To add to the list of inconclusive research findings, evidence regarding the role of perceived safety, maintenance and aesthetics in relation to physical activity outcomes is mixed [18,47,49,50,56]. Taken as a whole, our results combined with other studies highlight the need to further investigate the role and importance of perceived and objective measures to inform intervention development related to physical activity promotion.

The objective PEAT audits indicated numerous high quality walking trails in Hattiesburg, Mississippi. The number, excellence, and proximity of walking trails to residents' homes are conflicting to several studies that indicate a shortage of physical activity resources (e.g. parks and walking trails) in low income communities [22-24]. Rather, our study parallels those by Abercrombie and colleagues, which did not find deprivation of recreation facilities among low-income and high-minority neighborhoods in Maryland [20]. In our study, the availability and objective quality of trails rated generally high; however, evidenced by findings that the average use was 1.5 (S.D = 1.6) times per week and about 44% of respondents used the trails less than 0.5 times/week, overall trail use was relatively low. This is particularly noteworthy given that walking was the most preferred mode of physical activity for 61% of respondents, and that fewer respondents met PA recommendations by walking (35%) as compared to meeting PA recommendations (57%). Since 'meeting PA recommendations by walking' is defined in this study as walking 5 or more days a week for at least 30 minutes [38,39], it is conceivable that many of those indicating walking as a preferred activity walked less than the defined amount. Further, some may have chosen other walking locations besides trails for convenience (e.g. neighborhood streets), for protection from the weather (e.g. indoor facilities like a mall), or for other reasons that were not explored in this study.

Unlike prior findings, we did not find that trail use varied by demographic characteristics such as gender, age, race, or socioeconomic level [48]. Importantly, our study provides benchmark data related to health and physical activity patterns as well as baseline data for tracking future environmental changes. Based on self-reported height and weight, 70% of respondents were classified as overweight and obese. These rates far exceed national averages and HP 2020 goals, indicating that efforts to promote walking and other physical activity behaviors remain a high priority in Hattiesburg. Although reporting on quality indicators of individual trails was beyond the scope of this paper, individual trail characteristics also provide important data for specific improvements that are needed at each walking trail.

The evidence-based Guide to Community Preventive Services recommends four key strategies to promote physical activity in adults including enhanced access for physical activity along with informational outreach, community-wide campaigns, social-support interventions in community settings, and individually adapted health behavior change interventions [57,58]. In a simulated cost-effectiveness study, each of these four strategies offered substantial gains in quality-adjusted life years or good value for money, and no one strategy appeared more cost-effective than the others [59]. Our study provides critical formative data for program planning and implementation. For example, findings indicated that less investment is needed to build new walking trails or improve existing trails, and more efforts should be focused on maximizing the use of existing trails through community marketing campaigns and social support programming to decrease trail use barriers.

In the context of CBPR, key strategies are to build collaborative community-academic partnerships, develop a community's research skills, and promote data sharing and local dissemination of findings. This study signifies engagement of the community in an influential phase of research. Most of the community members hired and trained through this project have maintained leadership roles as peer-support coaches and assistance coaches in *H.U.B. City Steps *walking intervention [28,29]. Furthermore, to highlight and promote the characteristics, location, and amenities of each trail, results of the PEAT assessment were used to help develop a community resource guide for distribution via hard copy brochure and the *H.U.B. City Steps *website. Through on-going dissemination efforts, findings are being promoted through a written policy brief, and presentation to local government and civic groups. Within the CBPR framework, these lay methods of data dissemination are equally as important as hypothesis-testing and data-driven scientific dissemination. Creating a sense of community power and promoting community ownership of the individual, social, and environmental health problems and solutions will be key to improving the modifiable CVD risk factors and the long-term health of residents of Hattiesburg, Mississippi. Importantly, a recent paper that highlights opportunities to address disparities pertaining to recreation environments recommends the CBPR process to engage communities of color in research [13].

A few limitations should be considered when interpreting the findings of this study. First, a random sample promotes the greatest generalizability of study findings, yet was beyond the scope of this project. While use of the proportional sampling plan to match the targeted enrollment matrix for the larger CBPR walking intervention assures that our findings are sufficiently applicable to study population and intended use of data, the participants surveyed were much more active (i.e. 57% meeting physical activity recommendations) than what is typically reported by surveillance data (i.e. 37.5% of Mississippians meeting physical activity recommendations according to 2009 Behavioral Risk Factor Surveillance System data) [60]. It is not entirely clear if participants who met physical activity recommendations are overrepresented in our sample, as it was not our intent to recruit physically active participants or trail users, or if this discrepancy is a function of differences in methodology for reporting physical activity behaviors. While the validated IPAQ is one of the most frequently used estimates of self-reported physical activity [40], there are concerns for overestimation of physical activity with this instrument [42,61,62]. Regardless, the representativeness of our study population should be considered when interpreting the findings. Finally, scoring individual items into component scales and statistical modeling for items in the PEAT audit was challenging. While the PEAT proved to be a user friendly audit instrument and an appropriate tool for the broader objectives of this CBPR study[44,45], no known published papers have reported or modeled summary scales. Nonetheless, our methodological approach of creating standardized mean scores across four content areas is congruent with other standard approaches of modeling community audits, such as the Physical Activity Resource Assessment (PARA) instrument [47].

Future research efforts should address the limitations of this study. For examples, an objective indicator of physical activity, such as use of accelerometers, would provide a more accurate indication of physical activity patterns [63]. Second, future research should capitalize on understanding the broader built environment. Objective measurements of the built environment have made great advancements in recent years and gold standard measures are still emerging [11,16]. Of particular interest in Hattiesburg is to better understand the walkability and general environment for lifestyle physical activity, beyond just walking trails. For instance, GIS-derived measures could help better understand access to walking trail and recreational facilities, as well as street patterns, land-use mix, and population density [11]. While there is certainly room to utilize more sophisticated methods to advance the understanding of the physical activity patterns and environment in Hattiesburg, this study sufficiently informs intervention and policy development. Furthermore this study begins to fill an important void in the current literature as few identified papers have examined the built environment for physical activity in Mississippi [64], despite the fact that it consistently ranks among the least healthy states [3].

In conclusion, the modifiable risk factors related to CVD place a significant health burden on Mississippi's residents and health care systems. In addition to benefits for managing CVD risk factors, physical activity provides a constellation of health benefits including weight loss/maintenance, reduced cholesterol, stress management, and psychological well-being.

CBPR studies such as this one that engages communities and considers multiple levels of influence on physical activity are critical to identifying cost-effective and culturally relevant intervention approaches that have the potential for long-term sustainability, as well as the potential to inform local policies. Contrary to assumptions, numerous high quality walking trails/tracks were available within four city wards of a community with high minority population and low median income. In order to improve physical activity levels and decrease CVD risk factors, continued intervention efforts are needed to promote and maximize the use of existing trails, as well as improve residents' perceptions related to incivilities, safety, conditions of trail, and amenities of the walking trails. Furthermore, to best guide future programming efforts in Hattiesburg and similar communities, our findings should be mutually interpreted within the context of evidence-based physical activity recommendations and alongside approaches that have demonstrated cost-effectiveness potential for physical activity promotion [21,49,58,59].

## Competing interests

The authors declare that they have no competing interests.

## Authors' contributions

JZ and JH conceptualized and drafted the paper. Each author contributed to further development and revisions of the paper and assumed a unique role in execution of this research including: KY and JZ contributed to securing grant funding for the project; JZ and KZ conceptualized the study design, measurement, and evaluation; JH provided content and statistical expertise; and KZ and AS provided project and data management support. All authors read and approved the final manuscript.
